# Supplementary figures and images for: Natural variations of adolescent neurogenesis and anxiety predict the hierarchical status of adult inbred mice
Source: EMBO Rep. 2025 Jan 23;26(6):1440–56. doi: 10.1038/s44319-025-00367-y (PMC11933688; doi:10.1038/s44319-025-00367-y)

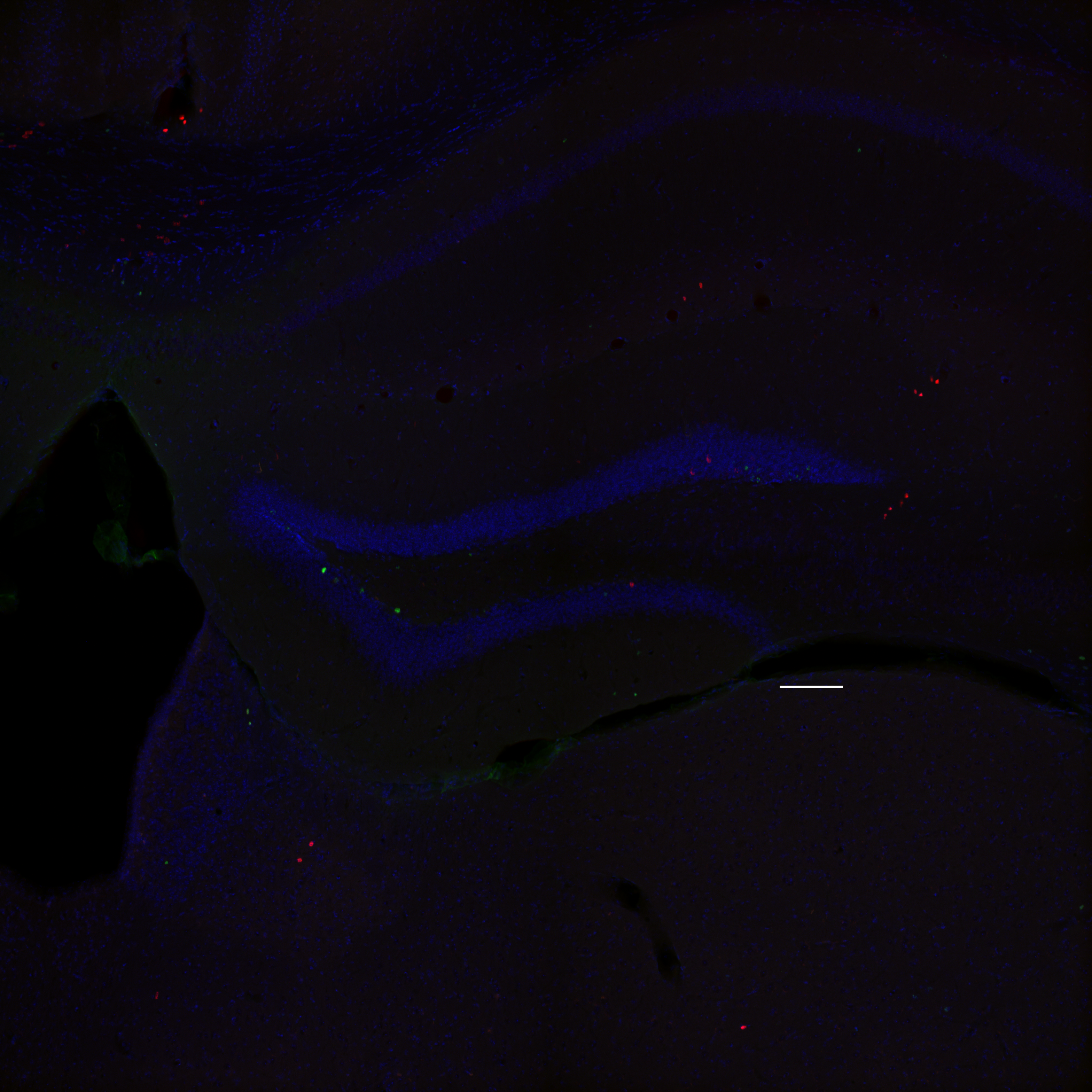

Supplement: Supplementary file 2 — Source data Fig. 1 [file 44319_2025_367_MOESM2_ESM.zip › Figure 1/1H/dominants.tif]

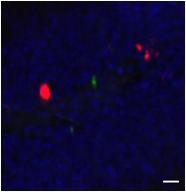

Supplement: Supplementary file 2 — Source data Fig. 1 [file 44319_2025_367_MOESM2_ESM.zip › Figure 1/1H/Inslet Sub CldU.tif]

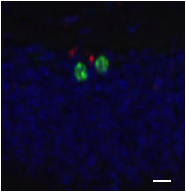

Supplement: Supplementary file 2 — Source data Fig. 1 [file 44319_2025_367_MOESM2_ESM.zip › Figure 1/1H/Inslet Sub.tif]

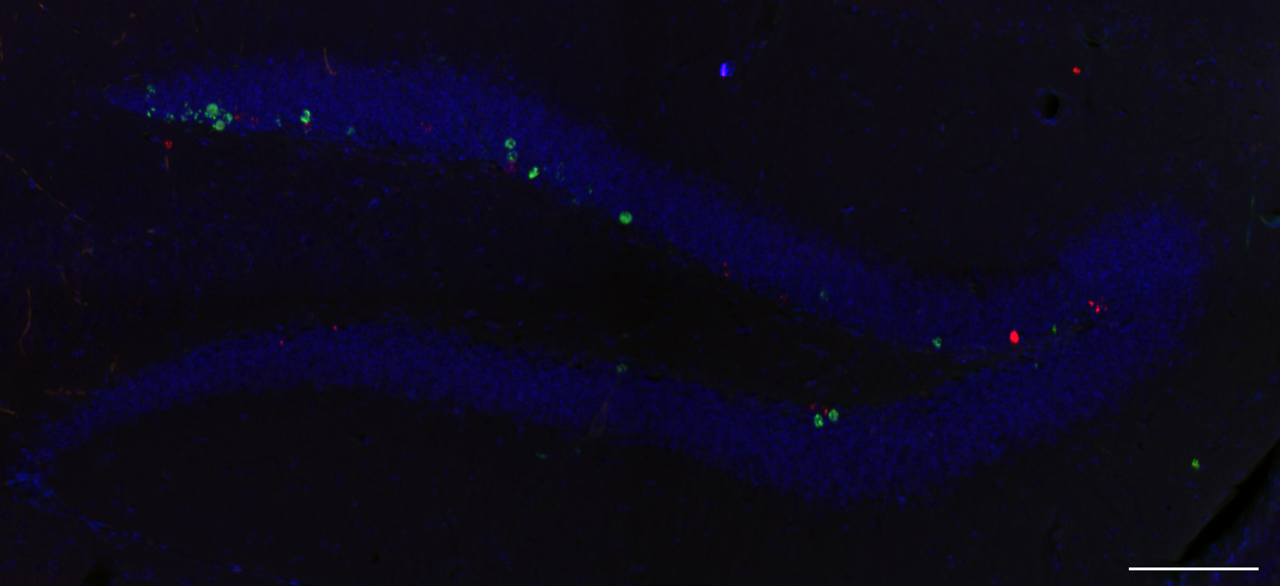

Supplement: Supplementary file 2 — Source data Fig. 1 [file 44319_2025_367_MOESM2_ESM.zip › Figure 1/1H/Sub.tif]

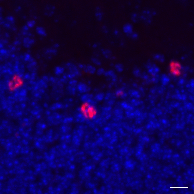

Supplement: Supplementary file 2 — Source data Fig. 1 [file 44319_2025_367_MOESM2_ESM.zip › Figure 2/2B/BrdU.tif]

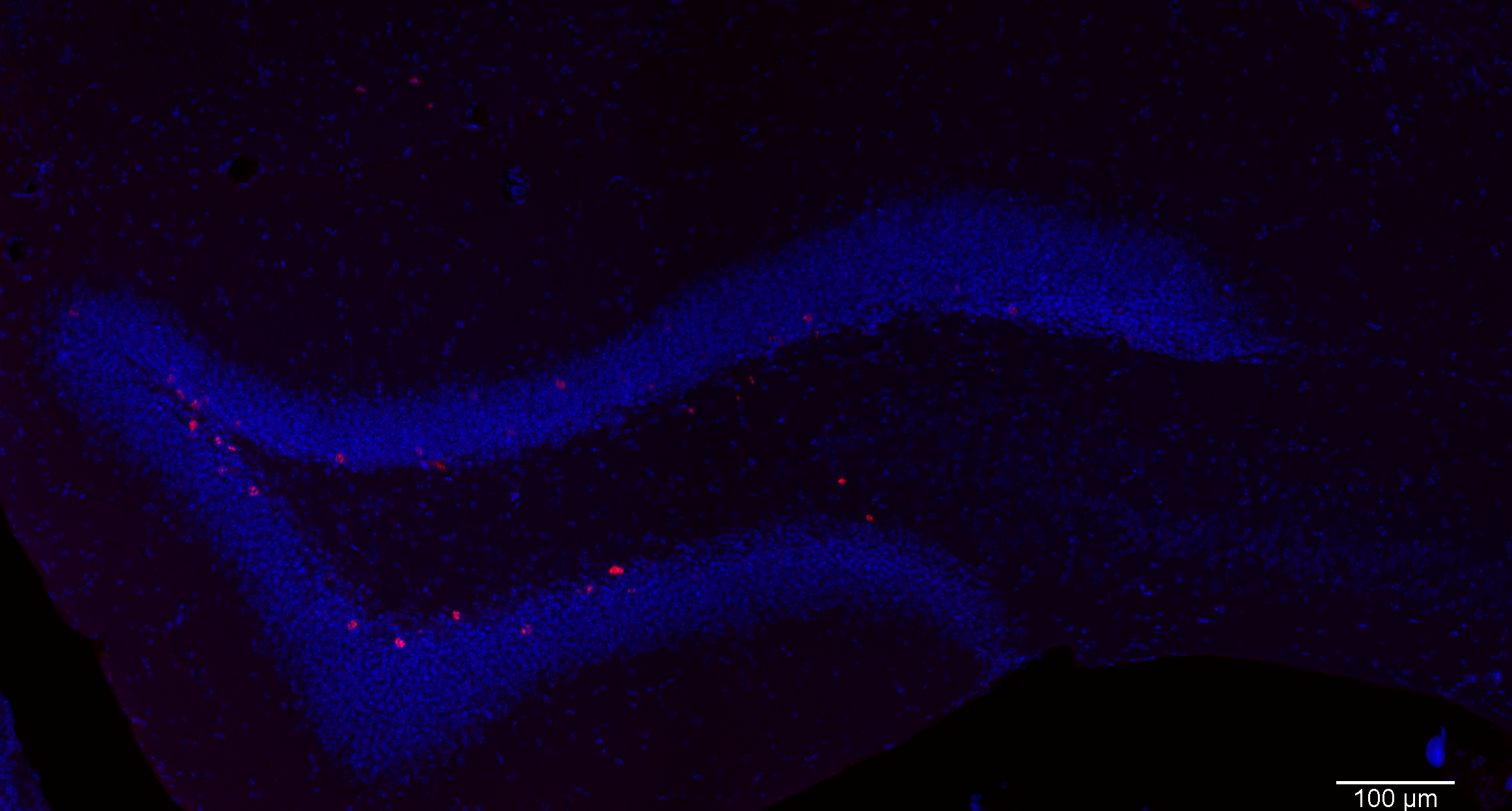

Supplement: Supplementary file 2 — Source data Fig. 1 [file 44319_2025_367_MOESM2_ESM.zip › Figure 2/2B/Inslet BrdU.tif]
