# Supplementary material for: Natural variations of adolescent neurogenesis and anxiety predict the hierarchical status of adult inbred mice
Source: EMBO Rep. 2025 Jan 23;26(6):1440–56. doi: 10.1038/s44319-025-00367-y (PMC11933688; doi:10.1038/s44319-025-00367-y)
Supplement: Supplementary file 6 — Expanded View Figures [file 44319_2025_367_MOESM6_ESM.pdf]

Expanded View Figures

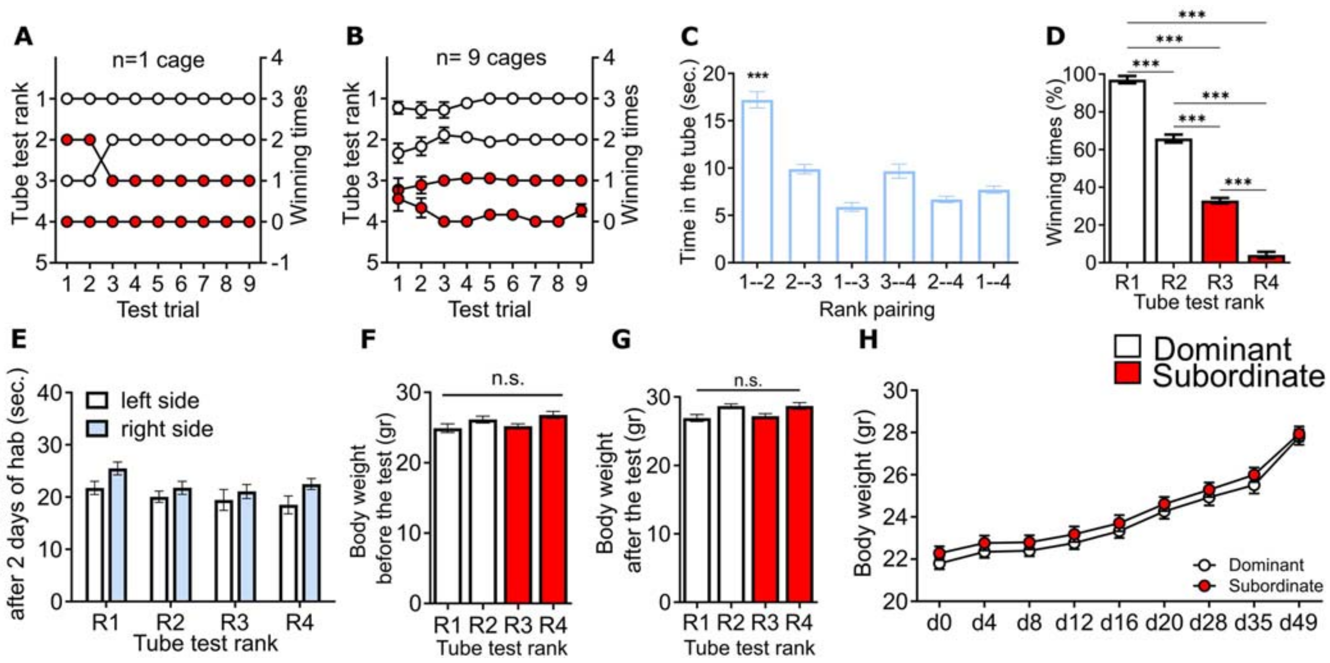

Figure EV1. Hierarchical rank assessed via a social confrontation tube test.

(**A**) Example of tube test ranks and winning times over trials for one cage ($n = 1$ cage). (**B**) Average ranks across nine cages over 9-day trials ($n = 9$ cages). (**C**) Time spent in the tube by rank pairings ($F(5,48) = 49.69$, $p < 0.001$, one-way ANOVA, $n = 9$ cages per pairing). (**D**) Winning percentages by rank after 9 days of confrontations ($F(3,42) = 494.6$, $p < 0.0001$, one-way ANOVA, $n = 9$ cages per pairing). (**E**) Average time spent in the tube during the 2-day habituation phase by final rank (interaction: $F(3,64) = 0.41$, $p = 0.7442$; rank effect: $F(3,64) = 2.44$, $p = 0.0719$; side effect: $F(1,64) = 7.787$, $p = 0.0983$; two-way ANOVA, $n = 9$ cages/group). (**F**, **G**) Body weight by rank before (**F**) and after (**G**) the tube test (before: $t34 = 0.89$, $p = 0.376$, unpaired t-test R1-R2 vs. R3-R4, two-tailed, $n = 18$ mice/group; after: $t34 = 0.30$, $p = 0.761$, unpaired t-test R1-R2 vs. R3-R4, two-tailed, $n = 18$ mice/group). (**H**) Body weight evolution in dominant versus subordinate mice. Histograms show average ± SEM; $^{***}p < 0.001$; ns = not significant. Source data are available online for this figure.

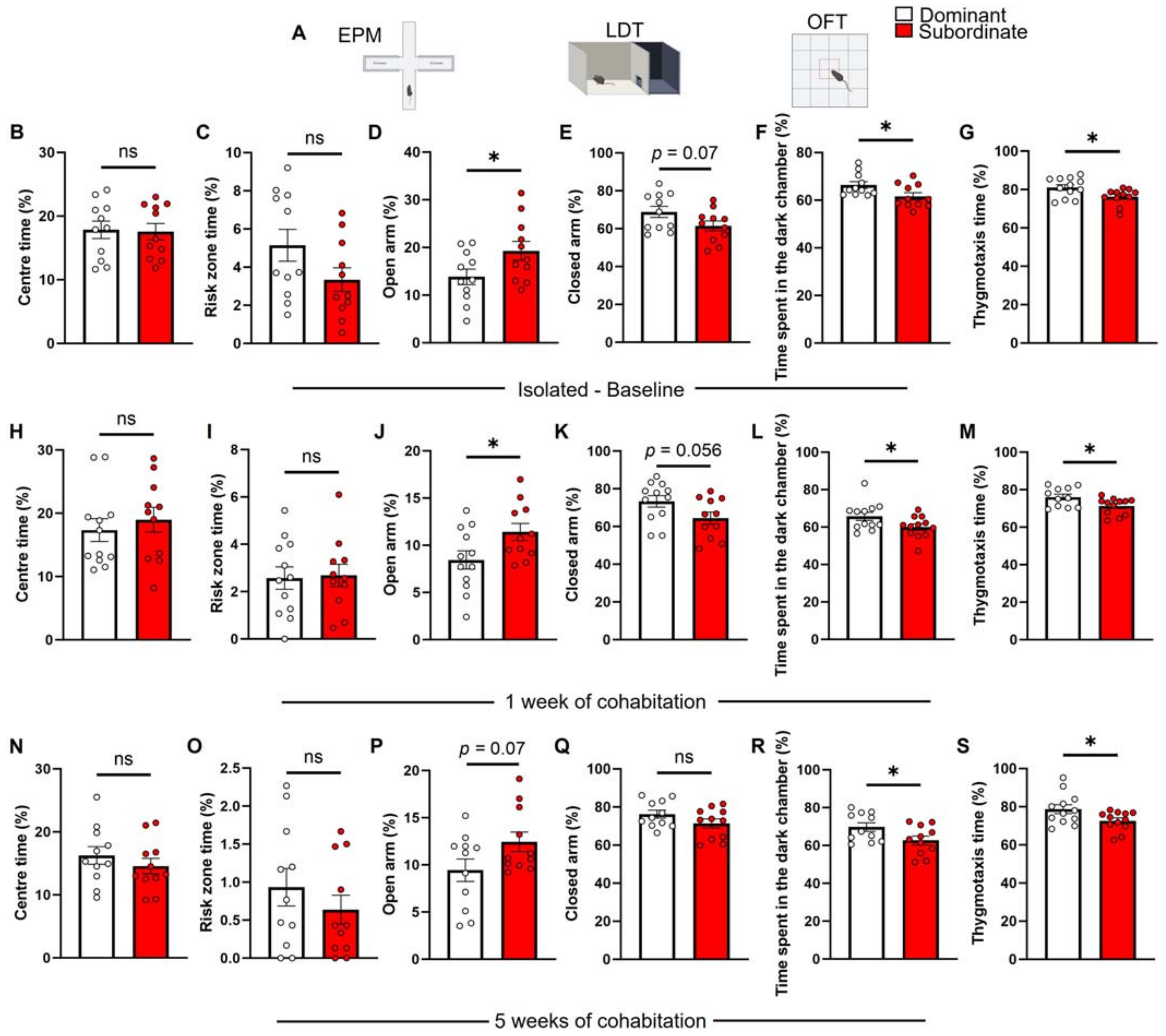

Figure EV2. Anxiety measures in dominant and subordinate individuals.

(**A**) Diagram of anxiety tests conducted. (**B–E**) Baseline behaviors in an EPM expressed as a percentage of time spent in the: (**B**) Center zone ($t20 = 0.156$, $p = 0.823$; unpaired t-test, two-tailed; $n = 11$/group). (**C**) Risk zone ($t20 = 1.733$, $p = 0.098$; unpaired t-test, two-tailed; $n = 11$/group). (**D**) Open arm ($t20 = 2.123$, $p = 0.046$; unpaired t-test, two-tailed; $n = 11$/group). (**E**) Closed arm ($t20 = 1.890$, $p = 0.073$; unpaired t-test, two-tailed; $n = 11$/group). (**F**) Percentage of time spent in the dark chamber in a LDT ($t20 = 2.29$, $p = 0.033$; unpaired t-test, two-tailed; $n = 11$/group). (**G**) Percentage of time in thigmotaxis in an OFT ($t20 = 2.44$, $p = 0.023$; unpaired t-test, two-tailed; $n = 12$ dominants, $n = 11$ subordinates). (**H–K**) Behavior in an EPM after one week of cohabitation expressed as a percentage of time spent in the: (**H**) Center zone ($t21 = 0.618$, $p = 0.543$; unpaired t-test, two-tailed; $n = 12$ dominants, $n = 11$ subordinates). (**I**) Risk zone ($t21 = 0.177$, $p = 0.861$; unpaired t-test, two-tailed; $n = 12$/group). (**J**) Open arm ($t21 = 2.231$, $p = 0.037$; unpaired t-test, two-tailed; $n = 12$/group). (**K**) Closed arm ($t21 = 2.021$, $p = 0.056$; unpaired t-test, two-tailed; $n = 12$/group). (**L**) Percentage of time spent in the dark chamber in a LDT after one week of cohabitation ($t22 = 2.093$, $p = 0.048$; unpaired t-test, two-tailed; $n = 12$/group). (**M**) Percentage of time in thigmotaxis in an OFT after one week of cohabitation ($t21 = 2.391$, $p = 0.026$; unpaired t-test, two-tailed; $n = 11$ dominants, $n = 12$ subordinates). (**N–Q**) Behavior in an EPM after one week of cohabitation expressed as a percentage of time spent in the: (**N**) Center zone ($t20 = 0.911$, $p = 0.373$; unpaired t-test, two-tailed; $n = 11$/group). (**O**) Risk zone ($t20 = 0.949$, $p = 0.354$; unpaired t-test, two-tailed; $n = 11$/group). (**P**) Open arm ($t20 = 1.901$, $p = 0.072$; unpaired t-test, two-tailed; $n = 11$/group). (**Q**) Closed arm ($t20 = 1.545$, $p = 0.138$; unpaired t-test, two-tailed; $n = 11$/group). (**R**) Percentage of time spent in the dark chamber in a LDT after five weeks of cohabitation ($t20 = 2.164$, $p = 0.043$; unpaired t-test, two-tailed; $n = 11$/group). (**S**) Percentage of time in thigmotaxis in an OFT after five weeks of cohabitation ($t22 = 2.188$, $p = 0.04$; unpaired t-test, two-tailed; $n = 12$/group). Histograms show average ± SEM. *$p < 0.05$; ns = not significant; n = number of animals. Source data are available online for this figure.

© The Author(s)

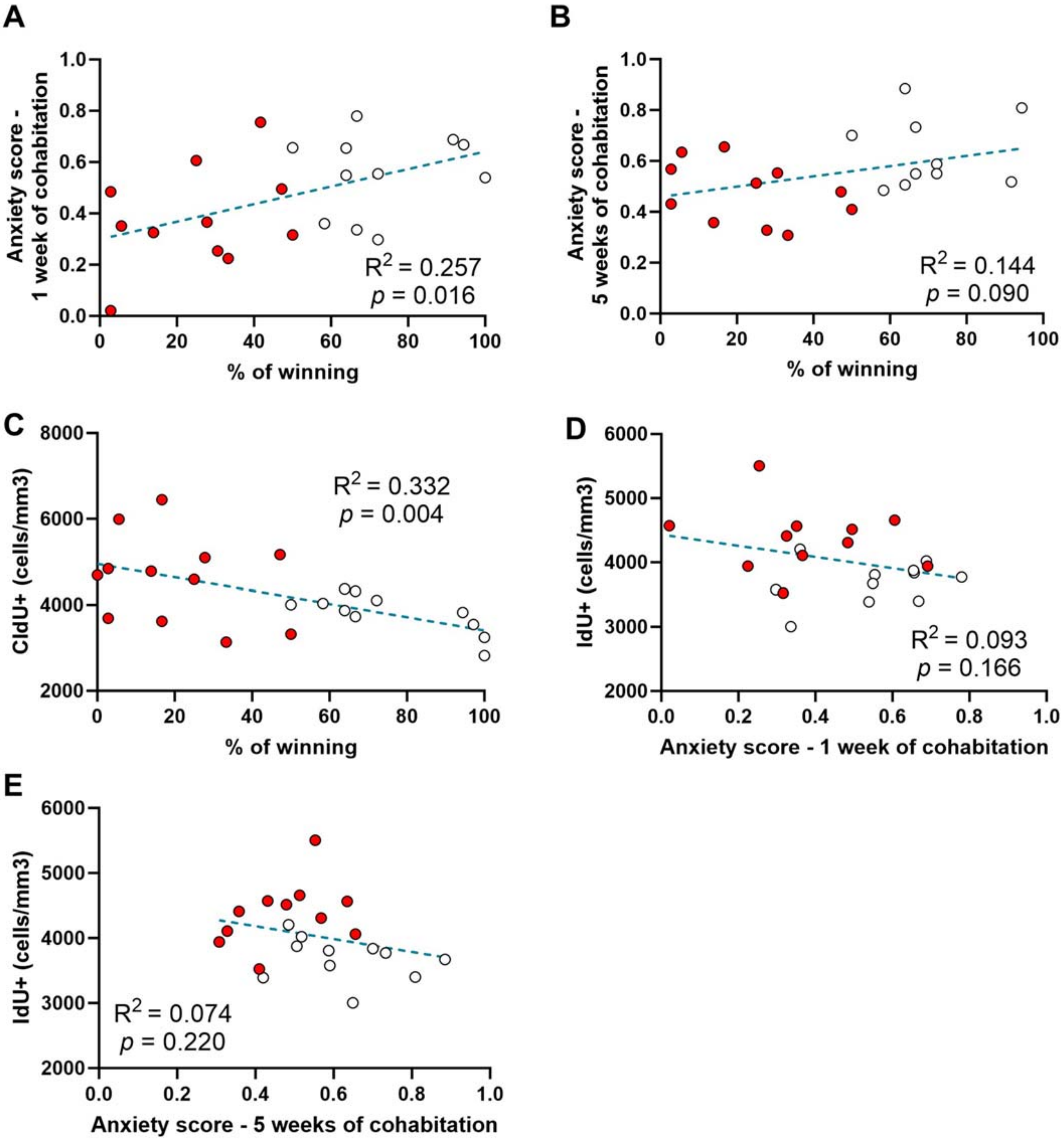

Figure EV3. Correlations between AHN, anxiety, and social dominance.

(**A**, **B**) Correlations between anxiety and percentage of winning during the SCTT after one week of cohabitation (**A**) and five weeks of cohabitation (**B**). (**A**, $R^2 = 0.257$, $p = 0.016$, simple linear regression, $n = 11$ mice per group. **B**, $R^2 = 0.144$, $p = 0.090$, simple linear regression, $n = 11$ subordinates, $n = 10$ dominants). (**C**) Correlation between the number of CldU-positive cells and percentage of winning during the SCTT ($R^2 = 0.332$, $p = 0.004$, simple linear regression, $n = 12$ subordinates, $n = 11$ dominants). (**D**, **E**) Correlation between the number of IdU-positive cells and the anxiety level after one week (**D**) and five weeks of cohabitation (**E**) (**D**, $R^2 = 0.093$, $p = 0.166$, simple linear regression $n = 11$ subordinates, $n = 10$ dominants. **E**, $R^2 = 0.074$, $p = 0.220$, simple linear regression, $n = 11$ mice per group). Blue line shows the linear regression. Source data are available online for this figure.

© The Author(s)

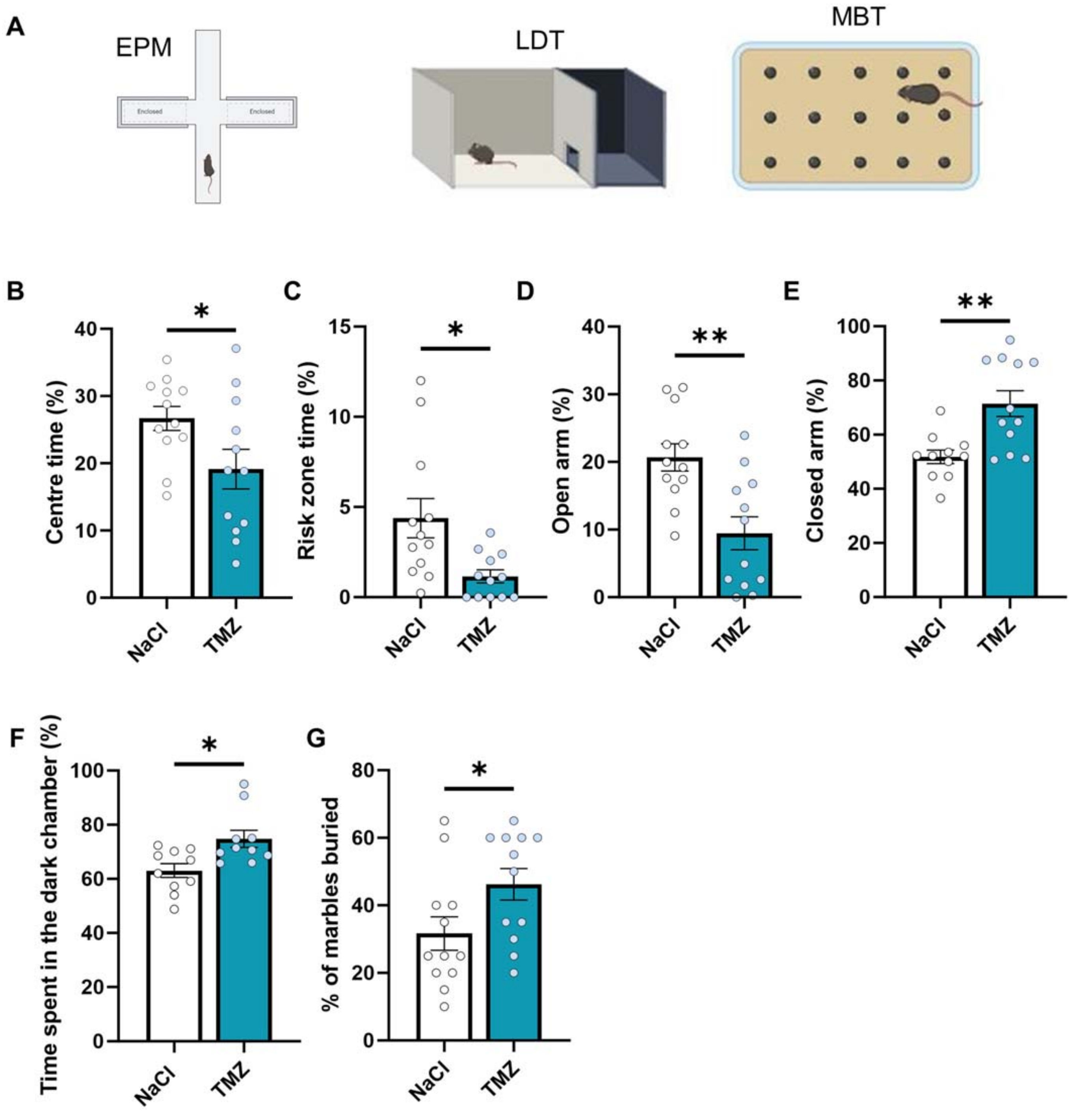

Figure EV4. Effect of TMZ on anxiety after group formation.

(**A**) Graphic representation of the behavioral tests used to assess anxiety. (**B–E**) Evaluation of several behaviors during EPM: percentage of time spent in the center (**B**), in risk zone (**C**), in the open arm (**D**) and in closed arm (**E**) (**B**, $t22 = 2.193$, $p = 0.039$, unpaired t test, two-tailed, $n = 12$ mice per group. **C**, $t22 = 2.813$, $p = 0.0101$, unpaired t test, two-tailed, $n = 12$ mice per group. **D**, $t22 = 3.556$, $p = 0.0018$, unpaired t test, two-tailed, $n = 12$ per group. **E**, $t21 = 3.553$, $p = 0.0019$, unpaired t test, two-tailed, $n = 12$ mice per TMZ, $n = 11$ per NaCl). Percentage of time in the dark chamber in LDT (**F**) and of marbles buried in MBT (**G**) (**F**, $t18 = 2.87$, $p = 0.01$, unpaired t test, two-tailed, $n = 10$ mice per group. (**G**), $t22 = 2.15$, $p = 0.042$, unpaired t test, two-tailed, $n = 12$ mice per group). Histograms show average ± SEM, $^{*}p < 0.05$, $^{**}p < 0.01$. Source data are available online for this figure.

 © The Author(s)

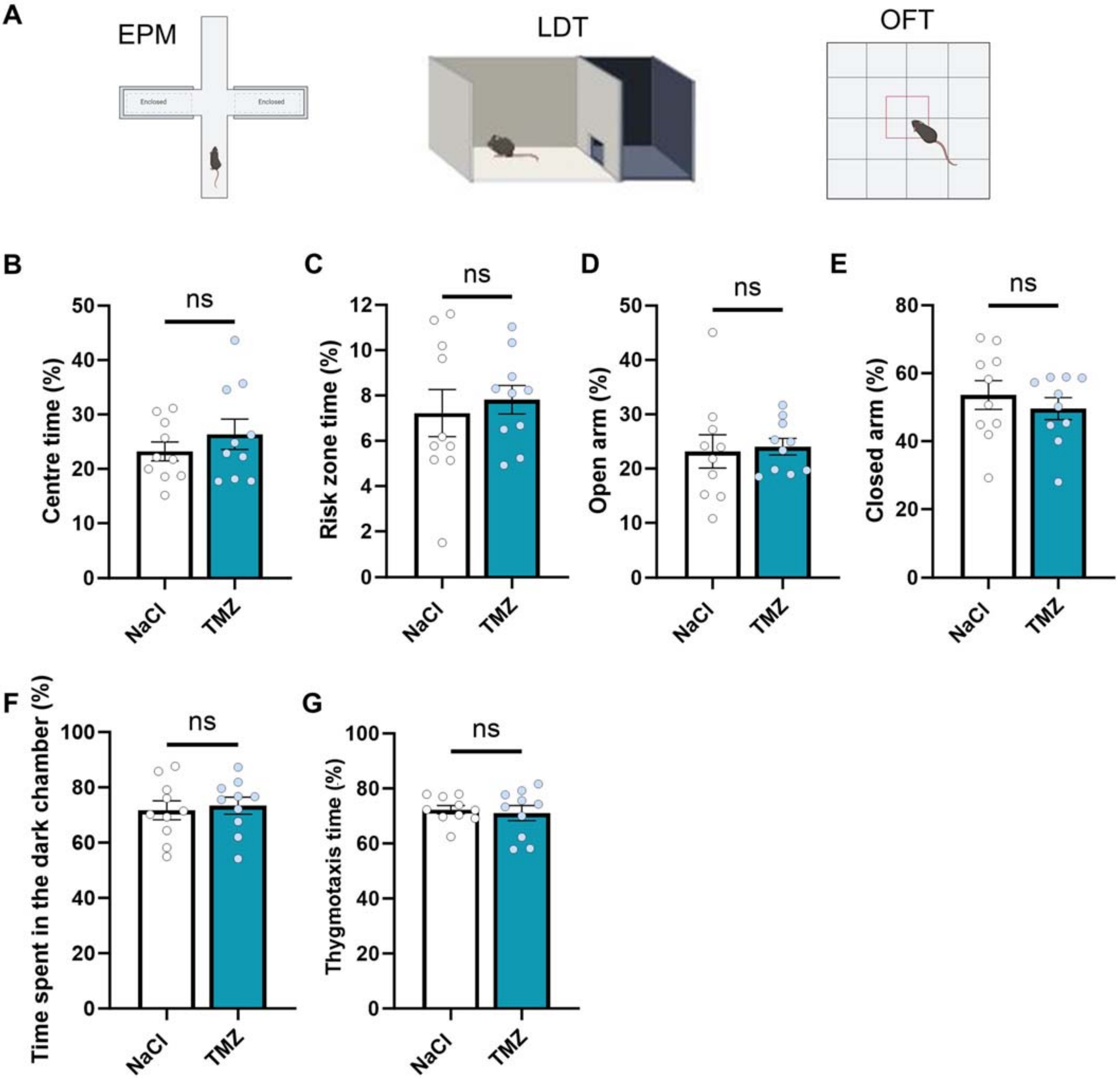

Figure EV5. Effect of TMZ on anxiety before group formation.

(**A**) Graphic representation of behavioral tests used to assess anxiety. (**B–E**) Evaluation of several behaviors during EPM: percentage of time spent in the center (**B**), in risk zone (**C**), in the open arm (**D**) and in closed arm (**E**) (**B**, t18 = 0.956, $p = 0.3514$, unpaired t test, two-tailed, $n = 10$ mice per group. **C**, t18 = 0.485, $p = 0.6334$, unpaired t test, two-tailed, $n = 10$ mice per group. **D**, t18 = 0.259, $p = 0.7986$, unpaired t test, two-tailed, $n = 10$ mice per group. **E**, t18 = 0.757, $p = 0.4587$, unpaired t test, two-tailed, $n = 10$ mice per group). Percentage of time spent in the, in the dark chamber in LDT (**C**) and in thygmotaxis during OFT (**D**) (**B**, t18 = 0.75, $p = 0.458$, unpaired t test, two-tailed, $n = 10$ mice per group; **C**, t18 = 0.36, $p = 0.720$, unpaired t test, two-tailed, $n = 10$ mice per group; **D**, t18 = 0.40, $p = 0.690$, unpaired t test, two-tailed, $n = 10$ mice per group). Histograms show average ± SEM, ns = not significant. Source data are available online for this figure.

© The Author(s)
